# Age-based stereotypes in hiring and training: how digitalization and labor shortages reshape employer decisions

**DOI:** 10.1007/s10433-026-00936-7

**Published:** 2026-07-30

**Authors:** Philipp Heyer, Wiebke Schmitz, Kathrin Weis

**Affiliations:** 1 Federal Institute for Vocational Education and Training, Bonn, Germany; 2 Federal Institute for Occupational Safety and Health, Berlin, Germany

**Keywords:** Age-Based Stereotypes, Digitalization, Labor Shortages, Vocational Education, BIBB Training Panel

## Abstract

Demographic aging contributes to labor shortages and strains welfare systems across Europe, including Germany. In response, extending older workers’ labor market participation has become a growing policy concern. Yet, age-based stereotypes may hinder this goal by reducing their chances in personnel decisions like hiring and training. However, the introduction of digital technologies is reshaping work dynamics, while labor shortages—partly driven by demographic aging—are intensifying competition for skilled workers. This puts pressure on companies to upskill their workforce and secure qualified personnel, potentially limiting the scope for decisions based on stereotypes.

While age-based stereotypes have been widely studied, the organizational context—such as labor shortages and digitalization—has received less attention. This study analyzes representative employer data from the 2024 BIBB Training Panel in Germany to examine (1) how age-based stereotypes among employers are linked to personnel decisions regarding hiring and training of older workers and (2) how this association differs by perceived labor shortages and digitalization levels.

Using fractional-logit regression models (n = 1310), the results show that while age-based stereotypes matter, their influence might be moderated by structural conditions: Digitalization mitigates training decisions that disadvantage older employees. There is also some evidence indicating that labor shortages may reduce hiring bias. These findings indicate that age-based stereotypes and their consequences do not operate in isolation: They are shaped by structural constraints and opportunities. In times of labor shortages and digital transformation, policy should address age-based stereotypes and reposition older workers not as a burden but as an asset.

**Supplementary Information:**

The online version contains supplementary material available at 10.1007/s10433-026-00936-7.

## Introduction

Demographic aging poses a major challenge for Germany, which has one of the oldest populations and lowest fertility rates in Europe (Eurostat 2025). While early retirement once offered firms especially in coordinated market economies (CME) like Germany a way to shed older workers despite strong job protection, policy today focuses on active aging and extended working lives (Wübbeke 2013; Clemens 2010). Consequently, older people in Germany are increasingly participating in the labor market. The employment rate of 55- to 64-year-olds rose from 64% in 2013 to 75% in 2023 (Statistisches Bundesamt 2025). For a long time, early retirement in company HR policies relied on underlying stereotypical assumptions about declining performance and productivity at older ages (Bellmann et al. 2003). These stereotypical views manifest especially in employment contexts, for instance, when older individuals are disadvantaged in hiring processes or in access to training opportunities, thereby preventing the full utilization of their potential as skilled workers (Kessler and Warner 2022; Solem 2016; Furunes and Mykletun 2010).This is particularly problematic in the context of demographic change: In view of rising life expectancy, declining birth rates, and the resulting shortage of skilled labor, it is crucial to keep older people in the workforce—not least to ensure the long-term performance and stability of social security systems. Furthermore, given the changing nature of work toward greater flexibility and digitalization, it is essential to more actively include older employees in training (Meinolf 2015).

Companies play a key role in responding to the consequences of such societal developments. Their HR policy decisions have implications not only within the organization but also at the macro level (Karpinska et al. 2011). While previous studies have not directly linked labor market conditions and digitalization to stereotypes, there is reason to assume that organizational and contextual factors influence how companies approach marginalized groups (Midtbøen 2015; McMullin and Dryburgh 2011). A perceived shortage of skilled workers may lead to increased hiring of older employees because companies are increasingly forced to tap into all available talent pools to stay competitive. In such situations, practical needs often override age-related biases. Similarly, companies with a higher level of digitalization may offer more training to older employees, as the use of digital tools make learning more necessary but also more accessible.

However, previous research has revealed little about age-based stereotypes from the perspective of employers with regard to training and hiring of older workers and its interplay with societal trends. This study extends prior research through four distinct contributions. Firstly, the current body of research has mostly focused on the consequences of age-based stereotypes on hiring (e.g., employment status) and training of older workers from the perspective of the older workers themselves without taking the employer’s perspective into account (Naegele et al. 2025).

Secondly, while the vast majority of studies have examined how older workers adapt to new technologies, there is still a lack of research on how the level of digitalization within the workplaces shapes the influence of age-based stereotypes on hiring and training decisions (Komp-Leukkunen 2023; Harris et al. 2018a, b). Thirdly, although perceived labor shortages are expected to enhance employers’ receptiveness toward older workers, research remains limited on how these perceived shortages interact with underlying age stereotypes to influence hiring and training outcomes. This study fills both research gaps by examining how digitalization within the workplace and employer’s perceived labor shortage influences the relationship between age-based stereotypes and hiring and training of older workers. Fourth, as an advanced economy, Germany presents an interesting case due to its institutional setup, often cited as prototypical coordinated market economy in VoC literature. This framework provides strong employment protection and places a high value on firm-specific knowledge, leading to low labor mobility—particularly among older employees.

Taken together, this article addresses previous research gaps using the latest representative data from the 2024 wave of the BIBB Training Panel, which specifically targets respondents involved in or responsible for personnel decisions. We use factor analysis to capture age-based stereotypes more comprehensively and apply fractional regression and moderator analyses to address the following research question: *Do age-based stereotypes influence hiring and training decisions regarding older workers, and how does this relationship vary depending on the perceived shortage of skilled labor and the degree of workplace digitalization?* We find that age-based stereotypes affect both hiring and training decisions. However, their impact may be moderated by structural conditions: While digitalization mitigates training decisions that disadvantage older employees, the influence of labor shortages appears to reduce hiring bias, although the evidence is less conclusive. Robustness checks indicate differences by firm size: age-based stereotypes shape personnel decisions only in small firms (1–99 employees), suggesting that organizational size plays an important role in shaping the relevance of stereotype effects. The findings indicate that age-based stereotypes and their consequences do not operate in isolation; rather, they are shaped by structural conditions.

## Theoretical background and literature review

Personnel decisions can classically be described using rational choice theory. This involves weighing up the costs of hiring decisions and training of workers against their benefits (Coleman 1990). Personnel decisions are shaped by company-related factors as well as by expectations regarding potential employees’ productivity (Karpinska et al. 2013). Stereotypes can distort these decisions by biasing cost–benefit evaluations through inaccurate group-based assumptions (van Dalen et al. 2009). Age-based stereotypes often depict older workers as less productive or adaptable. Despite lacking an evidence base, such assumptions still shape personnel decisions in many organizations (Marcus et al. 2016; Fiske et al. 2002). Following Fiske et al. (2002), who divide stereotypes into two superordinate categories—competence and warmth—Marcus et al. (2016) describe three groups of prejudices against older workers: Warmth, performance and adaptability. Perceived as warm, their performance and adaptability are rated negatively. Thus, stereotypes about older employees, such as supposedly lower adaptability or performance, lead to potential costs for induction or further training being overestimated, while benefits in the form of increased productivity are underestimated (Komp-Leukkunen 2023; Lu et al. 2011). This argument aligns with the concept of statistical discrimination, in which perceived productivity depends on nonproductive characteristics, such as age, when decision-makers use stereotypes to inform their assessments (Büsch et al. 2009; Arrow 1973; Phelps 1972). Beyond the economic perspective, which views stereotypes partly as conscious decisions, social psychological approaches highlight unconscious processes, through which deeply rooted stereotypes can produce discriminatory behavior without individuals being aware of it (Fiske 1998).

Research shows that advancing age is associated with reduced interview invitations, promotion opportunities, and participation in training, partly due to age-based stereotypes (Neumark et al. 2019; Solem 2016; Lössbroek and Radl 2019; Canduela et al. 2012; Furunes and Mykletun 2010; Büsch et al. 2009; Lössbroek and Schippers 2025). Studies indicate that managers holding negative views of older employees or endorsing early retirement policies are less inclined to hire or retain older workers, (Tunney and Mulders 2022; Karpinska et al. 2013; Lu et al. 2011) while exhibiting more favorable attitudes toward younger employees (Zanibonia et al. 2019). The likelihood of receiving further training also decreases if supervisors perceive older workers as resistant to learning or hold negative believes about their learning orientation (van Vianen et al. 2011; Bellmann et al. 2006)). Negative managerial stereotypes also foster an ageist climate, indirectly affecting older workers’ productivity and employment (Kunze et al. 2013).

Thus, we hypothesize that less negative age-based stereotypes are associated with a higher share of older hires among new employees (H1) and greater training participation among older employees (H2).

### Context of personnel decisions

Rational cost–benefit assessments also depend on situational conditions. As circumstances shift, so do incentives and constraints, potentially altering what was once seen as the optimal decision (Karpinska et al. 2011; Coleman 1990). The Resource Dependency Theory (RDT) suggests that in times of environmental uncertainty—induced by resource scarcity or constant change, like labor shortages or digitalization—decision-makers adjust their organizations’ strategies to secure resources and mitigate uncertainty (Pfeffer and Salancik 1978). As companies are embedded in a complex network of external dependencies, they react to resource constraints with strategic adjustments (Scott and Davis 2007). Qualified personnel is considered a resource, and access to it influences organizational strategy (Ansmann et al. 2021; Pettigrew et al. 1992).

From the RDT perspective, availability of suitable workers influences hiring strategies of companies. In phases of sufficient labor supply, age-based stereotypes often persist due to limited pressure to adjust personnel strategies. However, labor shortages create environmental uncertainty (Pfeffer and Salancik 1978), prompting organizations to reassess resource strategies. This shift encourages more objective assessments of older workers by reducing the influence of stereotypes. It impacts hiring—since rejecting qualified candidates becomes costly—and training, as firms must invest more in existing staff. Neglecting older employees in upskilling efforts risks skill obsolescence and may jeopardize productivity when external replacements cannot be sourced externally.

Studies that argue from the perspective of the RDT have shown that access to human resources influences hiring strategies of organizations (Ansmann et al. 2021). Recently, researchers also started to investigate the extent to which employers (can afford to) discriminate might vary with supply and demand. This research mainly focuses on hiring discrimination against minorities and point in the direction of decreasing discrimination in situations of labor shortages (Mergener and Maier 2019; Midtbøen 2015; Baert et al. 2013) However, some results suggest no or rather low effects of labor shortages on hiring discrimination (Lippens et al. 2022; Mergener and Maier 2019; Goldberg et al. 2013). Studies on the participation of older employees in further training and skills shortages are rare. General research results on further training and skills shortages show ambivalent results, in which both positive and negative effects of skills shortages on the participation of companies in further training were found (Mohr et al. 2013; Gerhards et al. 2012; Düll and Bellmann 1998).

We hypothesize that anticipated skill shortages mitigate the negative impact of age-based stereotypes on both hiring decisions (H3a) and training participation (H3b) of older employees.

### Digital technologies in personnel decisions

The introduction of new technologies generally has a negative impact on employees’ human capital, as it can render existing qualifications obsolete while requiring new, yet undeveloped skills (Mauno et al. 2019; Hudomiet and Willis 2021; Bartel and Sichermann 1993; Becker 1975). This challenge is particularly pronounced for older employees, whose initial training lies further in the past (Behaghel et al. 2014; Montizaan et al. 2007). To maintain their productivity, continuous investment in human capital becomes essential. Consequently, technological change is often accompanied by increasing training participation rates (Müller 2025).

Older employees are frequently perceived as being less willing to adapt—partly because investing in their training may seem less economically viable due to their proximity to retirement (Daniel and Heywood 2007). However, the significance of the amortization period diminishes when technological advancements accelerate and knowledge becomes obsolete more rapidly (Bellmann et al. 2006). In such a dynamic environment, technological know-how and specific forms of human capital become crucial resources for organizations. At the same time, access to external human resources is diminishing, as increasingly advanced and specialized skills are required.

RDT (Pfeffer and Salancik 1978) suggests that companies adapt to such developments by investing in the human capital of their workforce, irrespective of age. On the one hand, current employees already possess a significant share of the required human capital, which merely needs to be updated. On the other hand, due to the continuous pace of technological change, even human capital acquired through external hiring is subject to rapid obsolescence, making ongoing upskilling indispensable in the long term. These situations of technological development also increase the value of investing in the continuous training of older employees.

Empirical findings suggest that training mitigates the negative effects of ICT adoption on older workers (Behaghel et al. 2014). Furthermore, research has shown that expected technological change can extend the working life of older employees by fostering training opportunities (Haegeland et al. 2007; Bartel and Sichermann 1993). From the RDT perspective (Pfeffer and Salancik 1978), technological change can be understood as a development that alters organizational resource needs, specifically, the demand for highly specialized and continuously updated human capital. When technological advancements necessitate continuous skill adaptation, ongoing training becomes essential for all employees, regardless of age. In highly technology-driven firms, excluding older employees from training initiatives may not be a viable strategy, as it could impair their productivity and ultimately undermine the competitive advantage sought through technological innovation (Hudomiet and Willis 2021; Mauno et al. 2019; Soja and Soja 2020).

While digital technologies can support the training of older employees, their hiring may be adversely affected. From the perspective of RDT, technological change increases organizational dependence on human resources that possess highly specialized technological skills and demonstrate a high degree of adaptability. Consequently, firms may prioritize candidates perceived to meet these criteria in order to mitigate resource uncertainty, potentially reinforcing age-based selection biases in external hiring processes, as the gap between the required and the actual human capital hold by older applicants is perceived to be larger than in less digitalized companies. Research has shown that potential training costs play a significant role in hiring decisions (Thurow 1975). Additionally, technologies play a significant role in the impact of stereotypes toward older employees. Employees in sectors that heavily rely on technologies are perceived as older much earlier, or as generally unsuitable for their positions, a trend that can be attributed to stereotypical views regarding the lower adaptability of older workers (McMullin and Dryburgh 2011; Goldberg et al. 2013). Research indicates that technological innovations negatively affect the hiring of older employees, and, if their human capital is not updated through training, can shorten their working lives (Battisti et al. 2023; Behaghel et al. 2014; Yashiro et al. 2021).

With regard to hiring, we hypothesize that a higher technology level within the workplace amplifies the link between employers’ age-based stereotypes and the hiring of older workers (H4a). Moreover, we assume that a higher technology level within the workplace cushions the link between employers’ age-based stereotypes and the training of older workers (H4b).

## Methods

### Data basis

The analysis is based on the 2024 survey wave of the BIBB Establishment Panel on Training and Competence Development (BIBB Training Panel) (Friedrich and Gerhards 2023; Friedrich and Lukowski 2023). The BIBB Training Panel is a representative annual survey, conducted since 2011, encompassing approximately 3500 with German establishments at least one employee subject to social insurance contributions. The survey primarily targets HR managers, business owners, or managing directors—individuals directly involved in or responsible for personnel decisions. Thus, the dataset provides valuable insights relevant to our research question. In addition to general company information, such as size and industry, the survey highlights key topics, including (further) training, with the 2024 wave also placing a specific focus on older employees—defined here as those aged 50 and above.

#### Dependent variables

This analysis comprises two dependent variables. (1) Hiring decisions were measured by the share of older employees among all hires the company made in 2023. Secondly, (2) employees’ participation in training was measured by the share of older employees who received further training among all older employees in the company. Hereby, the dataset provides information on employees’ participation in internal or external training measures that were fully or partially sponsored by the employer through time off or cost coverage and do not aim to acquire a formal certificate. The aim of these training courses is to adapt employees’ skills to current or future work requirements by updating their skills.

#### Work-related age-based stereotypes

Seven questions were asked about the attitude toward older employees. Three items were used to assess the performance and four to assess the adaptability of older employees. These items were derived from the Work-Related Age-Based Stereotypes (WAS) scale developed by (Marcus et al. 2016), which has been validated as a suitable instrument for analyzing perceptions of older employees. The original scale comprises three dimensions: Capability (7 items), Adaptability (7 items), and Warmth (6 items). Since the latter dimension was not included in the BIBB Training Panel, this study focuses solely on the first two. Respondents rated their agreement with statements about older employees using a 6-point Likert scale (1 = no agreement, 6 = strong agreement). Items describing older employees in favorable terms (e.g., performance capacity, adaptability, flexibility) were reverse-coded prior to scale construction, so that higher values on the resulting measure consistently indicate more negative work-related age-based stereotypes. Although this assessment reflects the personal perception of the individual interviewed on behalf of the organization, it is plausible to assume that it is informed by both experience and prevailing opinions within the company. Moreover, the views of decision-makers and leaders may indirectly influence the perceptions of other employees involved in personnel decisions through a trickle-down effect (Mulders et al. 2017). Table S1 presents the individual items related to the statements and their distribution.

To examine the dimensional structure, both an exploratory (EFA) and confirmatory (CFA) factor analysis were conducted (Tables S4 and S5, Figure S1). The EFA was conducted using an oblique (Oblimin) rotation to permit inter-factor correlations (Kline 2023). While the EFA suggested a unidimensional latent structure, the subsequent CFA, which compared a one- and two-factor model, supported the two-factor solution. Nevertheless, the fit indices for both CFA models produced comparable results, and the correlation between the two latent constructs was found to be highly significant. Given this strong interrelationship, conducting regression analyses with both factors simultaneously would pose conceptual and statistical issues (James et al. 2021). Consequently, a unidimensional solution was adopted for the subsequent regression analyses. However, the findings do not contradict the results of Markus`s et al. (2016) several factor solution. Even though our dataset only includes a subset of the original seven items per construct. Moreover, the Warmth dimension was not assessed in the BIBB Training Panel.

Based on the results, one factor was constructed and included in the analysis as an independent variable. The internal consistency of the scale was assessed using Cronbach’s alpha, which, at 0.701, indicates an acceptable level of reliability. By minimizing random measurement errors and capturing the multidimensional nature of ageism more effectively than a single variable, the construct enhances the robustness of the analysis.

#### Structural context: labor market situation and technology level

To assess the labor market situation, companies were asked whether they anticipate difficulties in filling vacancies over the next three years. Measuring the expectation of staffing challenges is relevant, as personnel decisions are often made with a forward-looking perspective. Companies anticipate future workforce needs and adjust their hiring and training strategies accordingly. The variable is coded with three categories: 1 if difficulties are expected for most or all positions, 2 if difficulties are expected for some positions, and 3 if no difficulties are expected.

Empirical studies on the adoption of new technologies suggest that adaptation effects are either occupation-specific (Frey and Osborne 2017) or industry-specific (Autor et al. 2003). While our dataset does not allow for a direct measurement of occupation-specific technology levels, industry effects can be accounted for when constructing the technology indicator. The dataset provides information on the use of 13 technologies in the company. Table S6 lists the 13 technology types differentiated in the BIBB Training Panel. The number of technologies used was aggregated for each company. A dichotomous variable was then created, which, based on the eight industry-specific mean values, indicates whether a company has an above-average (1) or below-average (0) level of technology relative to its industry. This methodology enables a precise comparison of the digital equipment of companies within the same sector. As the use of digital technologies varies considerably between industries, a cross-industry measurement risks classification error. By assessing technology levels within each industry, our approach minimizes these errors, providing a more nuanced benchmark (Lammers et al. 2023).

#### Control variables

The assessment of older employees within companies and the personnel decisions based on this assessment largely depend on their tasks and qualification levels. This allows for potential reverse causation in line with the contact hypothesis (Fasbender and Wang 2017), as training-motivated, skilled older employees may influence their evaluations and subsequent personnel decisions. To account for this aspect, the qualification level of older employees was classified into three categories along the job-level: whether they work in jobs with low-skilled, skilled, and high-skilled tasks. The number of employees in each category was weighted by factors of 1 for simple, 2 for qualified, and 3 for highly qualified tasks. The weighted values were then summed and divided by the total number of older employees in the company to derive a variable that reflects the distribution of qualification level among older employees within the company. This variable ranges from 1 (low-skilled tasks) to 3 (highly-skilled tasks). Additionally, the model incorporates company size, measured in four categories; sector, measured in eight categories; if the company provides apprenticeship training; and the share of older employees within the company. This share serves as an indicator of prior personnel decisions and the presence of this group within the company, which may influence how they are perceived, thereby capturing potential reverse causal paths (Fasbender and Wang 2017).

Two control variables are included in only one of the two models. The proportion of applications from older employees received by the company, categorized into three groups, is considered solely in the analysis of the hiring of older employees. The further training participation rate for employees under the age of 50 is included as a control variable in the analysis of the participation rate of older workers in training.

### Sample description

The same sample was used in both models to ensure direct comparability and to prevent potential distortions arising from differences in sample composition. The initial sample of wave 2024 included 3.620 observations. However, 95 observations lacked data on the assessment of older employees. In addition, a subset of the sample (1647 observations) did not contain information on the two dependent variables. This large share is explained by the fact that companies could only provide information on hiring and training older employees if they had hired or trained at least one employee, regardless of age. The analytical sample also includes only establishments that had either received applications from or hired at least one employee aged 50 or older, resulting in the exclusion of a further 224 cases. Moreover, 344 cases were excluded due to missing information on the confounders. As a result, the analytical sample comprises 1.310 establishments. Table S1 presents descriptive statistics, including the seven variables measuring attitudes toward older employees.

### Empirical strategy

As both dependent variables are shares, the use of linear regression models can lead to biased or inefficient estimators. Therefore, fractional-logistic regression models are used for the empirical analysis, addressing the issue that the values of both dependent variables range between 0 and 1 (Papke and Wooldridge 1996, 2008). This approach is a common method for analyzing fractional dependent variables (Adegbesan and Higgins 2011).

First, the regression models for the two dependent variables are estimated without interaction terms to analyze the main effects of the independent variables. Second, to examine how the assessment of the company’s labor market situation and the industry-specific technology level moderate the relationship between age-based stereotypes and the two independent variables, interaction terms are added to the models. For each dependent variable, two interaction terms with age-based stereotypes were computed: one with expected staffing problems and another with industry-specific technology level, resulting in a total of additional four regression models.

## Results

### Main effects

**Fig. 1:**
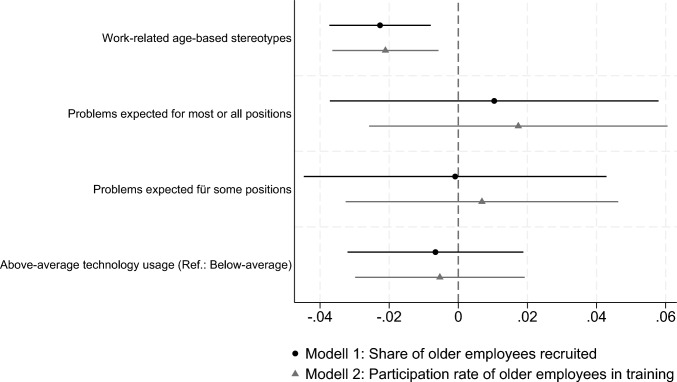
Average marginal effects on hiring and training older workers. Notes: Data from the BIBB Training Panel 2024; N = 1.310; Confidence intervals are set at 95%; both models include controls for sector, company size, West Germany, qualification level of older employees, the share of older employees in the company, and if the company provides apprenticeship training. Model 1 additionally controls for the share of applications from older workers, Model 2 for the participation rate of employees below 50 years of age in further training

Figure 1 presents the results of the fractional-logit regression models, reporting the average marginal effects (AME) of the explanatory variables on both dependent variables. Model 1 examines the share of older employees hired relative to all company hires in 2023. Model 2 analyzes the participation rate of older workers in training. In both models, age-based stereotypes are significantly correlated with the dependent variable. The more negatively organizational decision-makers perceive older employees, the lower the share of this group in the company’s hiring and in further training. Specifically, a one-point increase on the work-related age-based stereotype factor (ranging from -2.25 to 2.94) decreases the share of older workers hired by 2.3 percentage points (average 20%) and the share of older workers receiving training by 2.1 percentage points (average 32%). This confirms our prior expectations in H1 and H2. The company’s assessment of its labor market situation, on the other hand, is not significantly correlated with the dependent variables, neither with hiring decisions nor with further training of older employees. Similarly, technology use is not significantly correlated with the hiring or further training of older workers. Table S2 shows full regressions and models with reduced controls.

**Fig. 2:**
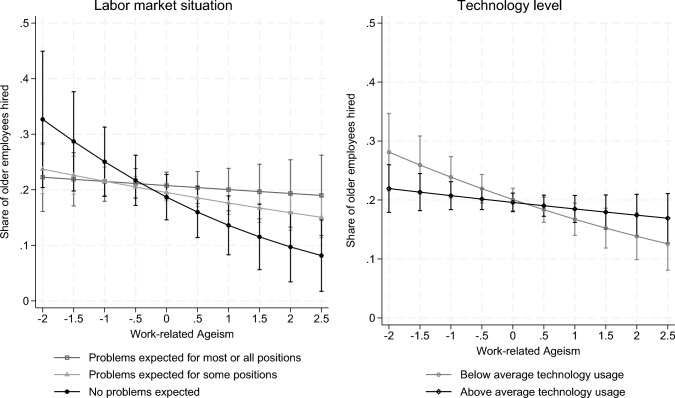
Conditional effects of work-related age-based stereotypes on the share of older employees hired. Notes: Data from the BIBB Training Panel 2024; N = 1.310; Confidence intervals set at 95%; both models include controls for sector, company size, West Germany, qualification level of older employees, the share of older employees in the company, the share of applications from older workers, and if the company provides apprenticeship training

In a second step, interaction terms were added to the regression models. Figure 2 illustrates the interactions for the share of older employees hired as dependent variable. The figure shows how the slope of Ageism varies across different levels of expected staffing problems and the company’s technology level. The interaction results show a weakly significant difference (10% level) between firms expecting staffing problems for most or all positions and those expecting no problems. In contrast, no significant differences are found for the intermediate category (problems expected for some positions). However, as shown in Fig. 2, the slopes (conditional marginal effects) of age-based stereotypes differ across labor market situations.^1^ The slope is largest when no staffing problems are expected (5.7 percentage point decrease) and decreases as expected staffing problems increase. Accordingly, the effect is smallest for firms expecting problems for most or all positions (0.7 percentage point decrease). Moreover, no significant conditional effect of age-based stereotypes on the share of older hires is found in this group, whereas the effect remains significant at the 5%- or 1%- level in the two categories with fewer or no expected staffing problems. Thus, the results are generally in the direction predicted by H3a, suggesting that in organizations anticipating staffing problems, age-based stereotypes among organizational decision-makers may have less influence on hiring older employees. However, the interaction is only weakly significant at the 10% level, providing limited empirical support for the hypothesis.

No significant interaction effect between technology use and age-based stereotypes can be identified. Thus, the level of technology does not influence the impact of organizational decision-makers’ stereotypes on the organization’s hiring decisions. This contradicts the stated expectations formulated in H4a.

**Fig. 3:**
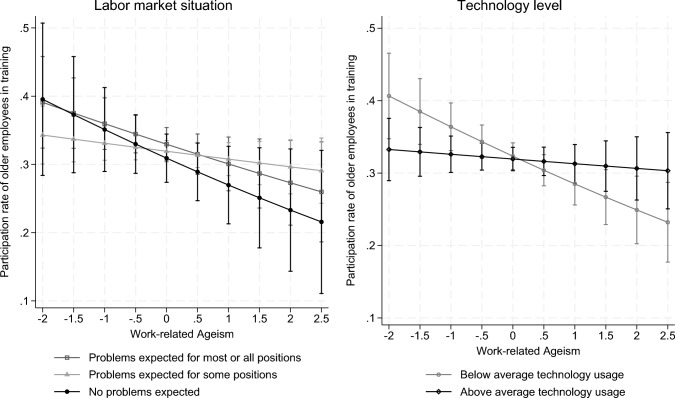
Conditional effects of work-related age-based stereotypes on the share of older employees trained. Notes: Data from the BIBB Training Panel 2024; N = 1.310; Confidence intervals set at 95%; both models include controls for sector, company size, West Germany, qualification level of older employees, the share of older employees in the company, the participation rate of employees below 50 years of age in further training, and if the company provides apprenticeship training

Figure 3 illustrates the results of the interaction terms, using the participation rate of older workers in training as the dependent variable.^2^ No significant interaction effect between expected staffing problems and age-based stereotypes is observed. Accordingly, the company’s assessment of its labor market situation does not appear to influence the correlation of age-based stereotypes of organizational decision-makers with training decisions concerning older employees. This finding contradicts the expectations of H3b. In contrast, the interaction term between technology level and age-based stereotypes is significant. Notably, the coefficient for age-based stereotypes on the participation rate of older workers in training is only significant in companies below the industry-standard technology level: A one-point increase on the age-based stereotypes scale decreases the share of older employees receiving training (average 32 percent) by 3.9 percentage points, whereas in firms with above-average technology usage the decline is not statistically significant, at 0.7 percentage points. This confirms our previous expectation of H4b.

### Heterogeneity analysis and robustness

#### Variation in technology specifications

As we used industry-specific averages of technology usage to define the firms’ level of technology usage in the main analysis (Chapter 4.1.), Tables S8 to S9.1 report results using an alternative measure of firms’ technology usage based on the overall rather than the industry-specific average. The findings are largely robust, with only minor differences in coefficient magnitudes.

To test whether the influence of stereotypes differs by type of technology, the main analysis variable is disaggregated into three separate variables: 3For ICT and Non-ICT, each dummy equals 1 if the firm’s usage exceeds the average based on a count of technologies used, and 0 otherwise. “AI usage” equals 1 if the firm uses at least one AI item, and 0 otherwise^4^.

Tables S10–S13.1 show a robust significant coefficient for work-related age-based stereotypes across all three specifications. The positive interaction term of above-average technology usage and age-based stereotypes for the share of older employees trained (Table S3) persists for ICT and Non-ICT usage but is insignificant for AI usage (Table S13). For the share of older employees hired, the interaction term turns significant for above-average Non-ICT usage, suggesting stronger stereotype effects on hiring in firms with lower-than-average Non-ICT usage (Table S12).

#### Firm size and sector

Tables S14–S16.1 present regression results by firm size and sector. The results reveal that the effect of age-based stereotypes on personnel decisions is largely driven by smaller firms with up to 99 employees, though the interaction term for training falls short of significance. These findings highlight the importance of firm size and the resulting heterogeneity across firms in shaping the effects of stereotypes.

Across sectors, interaction terms are insignificant for both hiring and training, and the significant conditional effects of age-based stereotypes are largely robust (Table S16,S16.1). Across both firm-size groups and sectors, labor shortage and technology usage remain insignificant for hiring older employees, and the same holds for training when firms are grouped by size. A sectoral divergence emerges for training, however: While above-average technology usage is associated with a lower training share in the service sector, the opposite holds in manufacturing, suggesting that the role of technological intensity in shaping training opportunities for older workers is meaningfully conditioned by sectoral context.

#### Share of older employees within firm

Personnel decisions may be shaped by the share of older employees within firms, both as a reflection of prior hiring behavior and as an indicator of exposure to older workers. While our main analysis includes this share as a control variable (Table S3), Table S17 tests whether it moderates the relationship between work-related age-based stereotypes and hiring decisions. However, the interaction term is insignificant, with comparable conditional effects across both subgroups (Table S17.1).

## Discussion and Conclusion

Drawing on the latest employer data from the 2024 BIBB Training Panel in Germany, this study explores how age-based stereotypes influence employers’ hiring and training decisions regarding older workers. It further examines how these effects vary based on (1) *perceived skill shortages* and (2) the *degree of digitalization in the workplace*.

We found that when employers hold fewer age-based stereotypes toward older workers, they *hire* and *train* a greater share of older employees. This aligns with prior research on age-based stereotypes in hiring (Tunney and Mulders 2022; Zanibonia et al. 2019; Lu et al. 2011) and training (van Vianen et al. 2011; Bellmann et al. 2006).

In line with RDT, our results suggest that expected labor shortages may encourage employers to hire older workers despite existing age-based stereotypes, although the statistical evidence is inconclusive. However, this pattern did not apply to training decisions, where age-based biases remained unchanged. This partly mirrors prior research on ethnic minority discrimination in tight labor markets (Baert et al. 2013; Mergener and Maier 2019), although, similar to our study, other research reports only weak or marginal effects (Lippens et al. 2022; Goldberg et al. 2013). One explanation for the missing effect on training is that, even amid labor shortages, older employees are still not seen as a viable solution, especially when age stereotypes persist. Additionally, older employees may withdraw from training themselves by internalizing such stereotypes, for instance, believing they are too old to learn (Naegele et al. 2025). Despite a weakened effect, the influence of stereotypes remains significant in contexts where staffing problems are expected for some positions—likely because, in uncertain decision-making situations involving external thus unfamiliar applicants, characteristics such as age continue to serve as a point of reference.

Another key finding is that higher workplace technology usage reduces age-based decisions that disadvantage older employees in access to training. This supports earlier research showing that technological change-induced demands for human capital lessen the impact of age stereotypes by making continuous skill development essential (Behaghel et al. 2014). Our results show that even in companies with negative views of older workers, high technology adoption is linked to consistently strong training efforts—confirming that digitalization can offset age bias by requiring ongoing human capital adaptation (Bellmann et al. 2006; Bartel and Sichermann 1993). Robustness checks also indicate differences by organizational size: the effects of age-based stereotypes are observed primarily in small firms (1–99 employees), suggesting that, beyond the technological conditions and labor shortages considered in this study, additional organizational factors play an important role in shaping the outcomes of age-based stereotypes. One possible explanation for this size effect lies in how decision-making processes vary with firm size. Small firms often lack formal HR departments and standardized procedures, leaving individual managers’ stereotypes greater scope to directly shape hiring and training decisions. Their training budgets also tend to be smaller and more flexible, allowing a single manager’s attitudes to shape outcomes more directly (Cardon and Stevens 2004).

The findings are noteworthy in that neither technology level nor labor shortages had direct effects; instead, their influence appears to be indirect—by shaping how age-based stereotypes affect decision-making. Focusing only on direct effects would likely underestimate the role of contextual factors, which, as this study shows, can moderate the impact of individual attitudes on personnel decisions. However, we found no evidence that *technology level* amplifies the link between *age-based stereotypes* and the *hiring* of older workers. This contradicts our assumption that digitalization intensifies age-based disadvantages in hiring. A possible explanation lies in the cross-sectional nature of our data, which captures a moment rather than a process of technological change. Moreover, retaining older workers may be less contentious than hiring unknown older employees. Digitalization may therefore encourage the upskilling of older employees already integrated into the organization, without necessarily leading to more inclusive external hiring. Other factors may also influence managerial decisions in times of crisis. Cost-avoidance strategies may be relevant: Hiring or retaining employees close to retirement represents a short-term investment compared to younger candidates. In periods of change induced by technological advancement, such long-term commitments may be considered problematic. While it appears to reduce stereotype effects in training, it does not do so in hiring. Further longitudinal research is needed to explore these dynamics (Komp-Leukkunen et al. 2022; König and Seifert 2022).

Taken together, our study suggests that age-based stereotypes are shaped by context: In times of labor shortages and digital transformation, policymakers should not only address age-based stereotypes directly but also create structural incentives promoting hiring and upskilling of older workers. Reframing older employees as a strategic asset during these times is essential for future-ready workforce planning.

### Limitations and future research

Our study has several limitations that should be addressed in future research. First, our findings are based on cross-sectional data and therefore represent only a snapshot in time. However, the consequences of broader societal trends—such as digitalization and labor shortages—can, as dynamic processes, only be fully understood through longitudinal analyses.

Second, the data only captured whether training was received, without accounting for its content or duration. Future research could therefore focus on the intensity and thematic scope of training programs.

Third, the mechanisms underlying the observed relationships warrant further investigation. For example, we relied on a reduced stereotype scale that focused on competence and adaptability but excluded the “warmth” dimension from the original WAS scale (Marcus et al. 2016), which may limit the scope of interpretation.

Fourth, while we find that organizational size shapes the effects of stereotypes, we cannot identify the underlying mechanisms. For instance, we cannot precisely determine the extent to which respondents are involved in personnel decision-making processes. In addition, differences by firm size may reflect not only organizational size itself, but also variation in decision formalization, external oversight, HR professionalization, and the degree of discretion in personnel decisions. Future studies should consider examining these and related factors.

Fifth, subsequent research should consider internalized age stereotypes among older workers, as self-doubt and perceived learning limits may reduce their engagement in training or job applications. Sixth, even though we account for the share of older employees in the firm and their skill levels, we can only partially address potential reverse causations arising from (positive) contact with older workers. This highlights longitudinal and individual-level analysis as a promising avenue for further research.

Seventh, while our results provide some indications of potential discrimination, they do not capture the precise mechanisms at play, leaving room for further investigation. Additionally, the continued employment of older workers allows firms to address short-term labor shortages while offering older employees significant opportunities to remain in the workforce, highlighting an important avenue for future study. Further, other incentives for hiring or retaining older employees should be considered. Employees approaching retirement, for instance, may be seen as a short-term, cost-effective solution. Such factors may function as competing considerations or even influence age-based stereotypes. Since our data could not capture these mechanisms, they remain an important direction for future research.

## Supplementary Information

Below is the link to the electronic supplementary material.

Supplementary file1 (DOCX 189 kb)

## Data Availability

The data used in this study stem from the 2024 wave of the BIBB Training Panel. At present, the dataset is not publicly available. However, it will be made accessible in the future via the Research Data Centre (FDZ) of the Federal Institute for Vocational Education and Training (BIBB). Once released, access can be requested through the FDZ at: https://www.bibb.de/en/1400.php

## Footnotes

1 Table S3 and Table S3.1 shows full regression models including interaction terms and conditional marginal effects.

2 Table S3 and Table S3.1 shows full regression models including interaction terms and conditional marginal effects.

3 Table S6 shows how the 13 technologies available in the BIBB Training Panel were assigned to the three groups.

4 See Table S7for descriptive statistics.
