# Supplementary material for: Age-based stereotypes in hiring and training: how digitalization and labor shortages reshape employer decisions
Source: Eur J Ageing. 2026 Jul 30;23(1):33. doi: 10.1007/s10433-026-00936-7 (PMC13457536; doi:10.1007/s10433-026-00936-7)
Supplement: Supplementary file 1 [file 10433_2026_936_MOESM1_ESM.docx]

Table S1: Descriptive statistics

|  | Mean | SD | Min | Max |
| --- | --- | --- | --- | --- |
| Share of older employees hired | 0.20 | 0.24 | 0.00 | 1.00 |
| Participation rate of older workers in training | 0.32 | 0.34 | 0.00 | 1.00 |
| Work-related age-based stereotypes* | 0.00 | 0.84 | -2.25 | 2.94 |
| Staffing problems expected in the next 3 years | 0.87 | 0.34 | 0.00 | 1.00 |
| Technology level | 0.57 | 0.50 | 0.00 | 1.00 |
| Qualification structure of older employees | 2.01 | 0.44 | 1.00 | 3.00 |
| Share of older employees within the organization | 0.35 | 0.16 | 0.02 | 0.89 |
| Company provides apprenticeship training | 0.61 | 0.49 | 0.00 | 1.00 |
| Participation rate of younger workers in training | 0.42 | 0.33 | 0.00 | 1.00 |
| **Sector** |  |  |  |  |
| Agriculture, Mining, and Energy | 0.07 | 0.26 | 0.00 | 1.00 |
| Manufacturing Industry | 0.23 | 0.42 | 0.00 | 1.00 |
| Construction Industry | 0.04 | 0.20 | 0.00 | 1.00 |
| Trade and Repair | 0.13 | 0.33 | 0.00 | 1.00 |
| Business-Related Services | 0.14 | 0.35 | 0.00 | 1.00 |
| Other Predominantly Personal Services | 0.12 | 0.33 | 0.00 | 1.00 |
| Medical Services | 0.14 | 0.34 | 0.00 | 1.00 |
| Public Sector and Education | 0.13 | 0.33 | 0.00 | 1.00 |
| Manufacturing and Industry | 0.34 | 0.48 | 0.00 | 1.00 |
| Service and Public Sector | 0.66 | 0.48 | 0.00 | 1.00 |
| **Company size** |  |  |  |  |
| 1 to 19 employees | 0.12 | 0.32 | 0.00 | 1.00 |
| 20 to 99 employees | 0.38 | 0.49 | 0.00 | 1.00 |
| 100 to 199 employees | 0.24 | 0.42 | 0.00 | 1.00 |
| 200 or more employees | 0.26 | 0.44 | 0.00 | 1.00 |
| Share of older applicants |  |  |  |  |
| Up to 20% of applicants aged 50 and older | 0.64 | 0.48 | 0.00 | 1.00 |
| 21% to 50% of applicants aged 50 and older | 0.31 | 0.46 | 0.00 | 1.00 |
| More than 50% of applicants aged 50 and older | 0.05 | 0.21 | 0.00 | 1.00 |
| **Assessment of older employees** |  |  |  |  |
| Older employees have high performance capacity | 4.19 | 1.11 | 1.00 | 6.00 |
| Productivity benefits from older employees | 2.63 | 1.23 | 1.00 | 6.00 |
| Older employees perform worse than younger workers | 1.98 | 1.12 | 1.00 | 6.00 |
| Older employees have great potential for development | 3.35 | 1.10 | 1.00 | 6.00 |
| Training older employees is not worthwhile | 2.25 | 1.24 | 1.00 | 6.00 |
| Older employees can quickly adapt to change | 3.64 | 1.06 | 1.00 | 6.00 |
| Older employees are often very flexible | 3.02 | 1.24 | 1.00 | 6.00 |

Notes: BIBB Training Panel 2024, N=1.310, unweighted, *Factor derived from the seven items for assessing older workers.

Table S2: Average Marginal Effects on hiring and training older employees

|  | **Share of older employees hired** | | | | **Share of older employees trained** | | | |
| --- | --- | --- | --- | --- | --- | --- | --- | --- |
|  | Model 1 |  | Model 2 |  | Model 3 |  | Model 4 |  |
| Age-based stereotypes | -0.024^**^ | (0.008) | -0.023^**^ | (0.007) | -0.022^**^ | (0.008) | -0.021^**^ | (0.008) |
| Problems expected for most or all positions | -0.001 | (0.025) | 0.010 | (0.024) | 0.014 | (0.022) | 0.017 | (0.022) |
| Problems expected for some positions | -0.019 | (0.023) | -0.001 | (0.022) | 0.001 | (0.020) | 0.007 | (0.020) |
| No problems expected |  |  |  |  |  |  |  |  |
| Below average technology usage |  |  |  |  |  |  |  |  |
| Above average technology usage | -0.019 | (0.013) | -0.007 | (0.013) | -0.012 | (0.012) | -0.005 | (0.013) |
| Qualification structure of older employees | -0.004 | (0.015) | -0.013 | (0.014) | 0.002 | (0.016) | 0.000 | (0.016) |
| Share of older employees within the organization | 0.215^**^ | (0.041) | 0.204^**^ | (0.040) | -0.042 | (0.045) | -0.038 | (0.045) |
| Up to 20% of applicants aged 50+ |  |  |  |  |  |  |  |  |
| 21% to 50% of applicants aged 50+ | 0.086^**^ | (0.014) | 0.093^**^ | (0.014) |  |  |  |  |
| More than 50% of applicants aged 50+ | 0.283^**^ | (0.043) | 0.264^**^ | (0.041) |  |  |  |  |
| Company provides apprenticeship training | -0.021^+^ | (0.013) | 0.002 | (0.015) | -0.022^+^ | (0.013) | -0.011 | (0.014) |
| 1 to 19 employees |  |  |  |  |  |  |  |  |
| 20 to 99 employees |  |  | -0.104^**^ | (0.032) |  |  | -0.009 | (0.026) |
| 100 to 199 employees |  |  | -0.131^**^ | (0.033) |  |  | -0.035 | (0.028) |
| 200 or more employees |  |  | -0.132^**^ | (0.033) |  |  | -0.033 | (0.028) |
| Manufacturing and Industry |  |  |  |  |  |  |  |  |
| Service and Public Sector |  |  | -0.007 | (0.013) |  |  | 0.005 | (0.013) |
| East Germany |  |  |  |  |  |  |  |  |
| West Germany |  |  | -0.015 | (0.016) |  |  | -0.013 | (0.015) |
| Participation rate of younger workers in training |  |  |  |  | 0.600^**^ | (0.012) | 0.595^**^ | (0.012) |

Notes: BIBB Training Panel 2024, N=1.310, Reported coefficients are average marginal effects; Standard errors in parentheses, ^+^ *p* < 0.10, ^*^ *p* < 0.05, ^**^ *p* < 0.01

Table S3: Regression Results Including Interaction Terms (Standardized Beta Coefficients)

|  | **Share of older employees hired** | | | | **Share of older employees trained** | | | |
| --- | --- | --- | --- | --- | --- | --- | --- | --- |
|  | Model 5 |  | Model 6 |  | Model 7 |  | Model 8 |  |
| Age-based stereotypes | -0.400^*^ | (0.157) | -0.237^**^ | (0.083) | -0.272^+^ | (0.162) | -0.258^**^ | (0.083) |
| Problems expected for most or all positions | 0.140 | (0.169) | 0.060 | (0.162) | 0.134 | (0.145) | 0.104 | (0.146) |
| Problems expected for some positions | 0.057 | (0.158) | -0.013 | (0.151) | 0.067 | (0.133) | 0.035 | (0.134) |
| No problems expected |  |  |  |  |  |  |  |  |
| Problems expected for most or all positions  # Age-based stereotypes | -0.353^+^ | (0.182) |  |  | -0.081 | (0.191) |  |  |
| Problems expected for some positions  # Age-based stereotypes | 0.267 | (0.168) |  |  | 0.195 | (0.174) |  |  |
| No problems expected # Age-based stereotypes |  |  |  |  |  |  |  |  |
| Below average technology usage |  |  |  |  |  |  |  |  |
| Above average technology usage | -0.051 | (0.087) | -0.032 | (0.086) | -0.036 | (0.082) | -0.026 | (0.082) |
| Below average technology usage  # Age-based stereotypes |  |  |  |  |  |  |  |  |
| Above average technology usage  # Age-based stereotypes |  |  | 0.160 | (0.102) |  |  | 0.215^*^ | (0.108) |
| Qualification structure of older employees | -0.076 | (0.095) | -0.088 | (0.095) | 0.006 | (0.107) | 0.005 | (0.107) |
| Share of older employees within the organization | 1.385^**^ | (0.269) | 1.374^**^ | (0.271) | -0.237 | (0.298) | -0.231 | (0.297) |
| 1 to 19 employees |  |  |  |  |  |  |  |  |
| 20 to 99 employees | -0.577^**^ | (0.165) | -0.588^**^ | (0.167) | -0.053 | (0.166) | -0.057 | (0.167) |
| 100 to 199 employees | -0.762^**^ | (0.172) | -0.768^**^ | (0.174) | -0.227 | (0.181) | -0.218 | (0.181) |
| 200 or more employees | -0.775^**^ | (0.170) | -0.784^**^ | (0.172) | -0.214 | (0.182) | -0.218 | (0.182) |
| Manufacturing and Industry |  |  |  |  |  |  |  |  |
| Service and Public Sector | -0.046 | (0.089) | -0.044 | (0.088) | 0.031 | (0.087) | 0.037 | (0.087) |
| East Germany |  |  |  |  |  |  |  |  |
| West Germany | -0.106 | (0.103) | -0.102 | (0.103) | -0.088 | (0.097) | -0.095 | (0.098) |
| Company provides apprenticeship training | 0.006 | (0.097) | 0.015 | (0.098) | -0.073 | (0.090) | -0.070 | (0.090) |
| Up to 20% of applicants aged 50+ |  |  |  |  |  |  |  |  |
| 21% to 50% of applicants aged 50+ | 0.609^**^ | (0.089) | 0.602^**^ | (0.089) |  |  |  |  |
| More than 50% of applicants aged 50+ | 1.422^**^ | (0.179) | 1.416^**^ | (0.182) |  |  |  |  |
| Participation rate of younger workers in training |  |  |  |  | 3.912^**^ | (0.145) | 3.909^**^ | (0.145) |
| Pseudo *R*^2^ | 0.0597 |  | 0.0591 |  | 0.2486 |  | 0.2491 |  |

Notes: BIBB Training Panel 2024, N=1.310, Standardized beta coefficients; Standard errors in parentheses, ^+^ *p* < 0.10, ^*^ *p* < 0.05, ^**^ *p* < 0.01

Table S3.1: Conditional effects of Age-based stereotypes

|  | Col1: Hiring  (#Labor shortage) | | Col2: Training  (#Labor shortage) | | Col3: Hiring  (#Tec) | | Col4: Training  (#Tec) | |
| --- | --- | --- | --- | --- | --- | --- | --- | --- |
| Problems expected for most or all positions | -0.007 | (0.014) | -0.029^+^ | (0.015) |  |  |  |  |
| Problems expected for some positions | -0.020^*^ | (0.009) | -0.012 | (0.010) |  |  |  |  |
| No problems expected | -0.057^**^ | (0.022) | -0.040^+^ | (0.024) |  |  |  |  |
| Below average technology usage |  |  |  |  | -0.036^**^ | (0.013) | -0.039^**^ | (0.013) |
| Above average technology usage |  |  |  |  | -0.011 | (0.009) | -0.007 | (0.010) |

Notes: BIBB Training Panel 2024, N=1.310, Standard errors in parentheses, ^+^ *p* < 0.10, ^*^ *p* < 0.05, ^**^ *p* < 0.01

Table S4: Results of the Confirmatory Factor Analyses

|  | 2-Factor model without covariance | | 2-Factor model allowing for covariance | | 1-Factor model | |
| --- | --- | --- | --- | --- | --- | --- |
| Older employees have high performance capacity |  |  |  |  |  |  |
| Capability | 1.00 |  | 1.00 |  |  |  |
| Ageism |  |  |  |  | 1.00 |  |
| Productivity benefits from older employees |  |  |  |  |  |  |
| Capability | -0.86^***^ | (0.109) | -0.95^***^ | (0.062) |  |  |
| Ageism |  |  |  |  | -0.98^***^ | (0.061) |
| Older employees perform worse than younger workers |  |  |  |  |  |  |
| Capability | -0.49^***^ | (0.064) | -0.52^***^ | (0.049) |  |  |
| Ageism |  |  |  |  | -0.54^***^ | (0.051) |
| Older employees have great potential for development |  |  |  |  |  |  |
| Adaptability | 1.00 |  | 1.00 |  |  |  |
| Ageism |  |  |  |  | -0.86^***^ | (0.056) |
| Training older employees is not worthwhile |  |  |  |  |  |  |
| Adaptability | 0.41^***^ | (0.068) | 0.45^***^ | (0.063) |  |  |
| Ageism |  |  |  |  | -0.42^***^ | (0.057) |
| Older employees can quickly adapt to change |  |  |  |  |  |  |
| Adaptability | 1.15^***^ | (0.104) | 0.96^***^ | (0.065) |  |  |
| Ageism |  |  |  |  | 0.81^***^ | (0.055) |
| Older employees are often very flexible |  |  |  |  |  |  |
| Adaptability | 1.03^***^ | (0.091) | 1.09^***^ | (0.080) |  |  |
| Ageism |  |  |  |  | -0.96^***^ | (0.063) |
| var(e.b_ageismus1) | 0.55^***^ | (0.087) | 0.61^***^ | (0.043) | 0.70^***^ | (0.038) |
| var(e.b_ageismus2) | 1.02^***^ | (0.074) | 0.96^***^ | (0.050) | 1.01^***^ | (0.048) |
| var(e.b_ageismus3) | 1.08^***^ | (0.047) | 1.08^***^ | (0.045) | 1.09^***^ | (0.045) |
| var(e.b_ageismus4) | 0.80^***^ | (0.047) | 0.76^***^ | (0.040) | 0.80^***^ | (0.038) |
| var(e.b_ageismus5) | 1.47^***^ | (0.059) | 1.45^***^ | (0.058) | 1.45^***^ | (0.058) |
| var(e.b_ageismus6) | 0.59^***^ | (0.050) | 0.72^***^ | (0.038) | 0.78^***^ | (0.037) |
| var(e.b_ageismus7) | 1.11^***^ | (0.056) | 1.02^***^ | (0.051) | 1.05^***^ | (0.049) |
| var(Capability) | 0.68^***^ | (0.095) | 0.62^***^ | (0.055) |  |  |
| var(Adaptability) | 0.40^***^ | (0.050) | 0.44^***^ | (0.045) |  |  |
| cov(Capability,Adaptability) |  |  | 0.43^***^ | (0.033) |  |  |
| var(Ageism) |  |  |  |  | 0.53^***^ | (0.047) |

Notes: BIBB Training Panel 2024, N = 1.310, Standard errors in parentheses, ^*^ *p* < 0.05, ^**^ *p* < 0.01, ^***^ *p* < 0.001

Table S5: Fit indices of the Confirmatory Factor Analyses

| **Fit statistic** | **2-Factor model without**  **covariance** | **2-Factor model allowing**  **covariance** | **1-Factor model** |
| --- | --- | --- | --- |
| Likelihood ratio |  |  |  |
| Chi² | 549,5 | 130,4 | 159,6 |
| p > Chi² | 0,00 | 0,00 | 0,00 |
| RMSEA | 0,171 | 0,083 | 0,089 |
| Information criteria: |  |  |  |
| AIC | 27.814,4 | 27397,3 | 27.424,5 |
| BIC | 27.923,1 | 27.511,2 | 27533,3 |
| Baseline comparison: |  |  |  |
| CFI | 0,619 | 0,916 | 0,896 |
| SRMR | 0,175 | 0,049 | 0,052 |

Notes: BIBB Training Panel 2024, N = 1.310

Figure S1: Scree plot of Eigenvalues from Exploratory Factor Analysis

Notes: BIBB Training Panel 2024, N =1.310

Table S6: Types of technologies in BIBB Training Panel 2024

| Digital technologies related to customer services | ICT |
| --- | --- |
| Digital technologies related to networking with suppliers/businesses | ICT |
| Human resources management technologies | ICT |
| New forms of communication and collaboration | ICT |
| Support for project-based and cross-organizational collaboration | ICT |
| Technologies for storing and processing large amounts of data | ICT |
| Specialized software and hardware for IT security | ICT |
| Technologies that enable innovative networking and automation | Non-ICT |
| More customized production technologies | Non-ICT |
| Digital devices worn by employees (wearables) | Non-ICT |
| Technology for autonomous transportation | Non-ICT |
| The use of AI and machine learning in physical work processes | AI |
| The use of AI and machine learning in non-physical work processes | AI |

Table S7: Descriptive statistics on different indicators on type of technology

|  | Mean | SD | Min | Max |
| --- | --- | --- | --- | --- |
| Above average technology level (industry-specific) | 0.57 | 0.50 | 0.00 | 1.00 |
| AI usage (0/1) | 0.18 | 0.38 | 0.00 | 1.00 |
| ICT usage (above average) | 0.61 | 0.49 | 0.00 | 1.00 |
| Non-ICT usage (excl. AI) (above average) | 0.33 | 0.47 | 0.00 | 1.00 |
| Above average technology level (overall) | 0.54 | 0.50 | 0.00 | 1.00 |

Notes: BIBB Training Panel 2024, N = 1.310

Table S8: Average Marginal Effects on hiring and training older workers (different indicator on technology level (above overall average))

|  | Col1: Hiring  (above average  technology usage  (overall average)) | | Col2: Training  (above average  technology usage  (overall average)) | |
| --- | --- | --- | --- | --- |
| Age-based stereotypes | -0.022^**^ | (0.007) | -0.021^**^ | (0.008) |
| Problems expected for most or all positions | 0.011 | (0.024) | 0.017 | (0.022) |
| Problems expected for some positions | -0.001 | (0.022) | 0.006 | (0.020) |
| No problems expected |  |  |  |  |
| Below average Tec usage (overall average) |  |  |  |  |
| Above average Tec usage (overall average) | 0.000 | (0.013) | -0.014 | (0.012) |
| Qualification structure of older employees | -0.013 | (0.014) | 0.001 | (0.016) |
| Share of older employees within the organization | 0.205^**^ | (0.040) | -0.042 | (0.045) |
| Up to 20% of applicants aged 50+ |  |  |  |  |
| 21% to 50% of applicants aged 50+ | 0.093^**^ | (0.014) |  |  |
| More than 50% of applicants aged 50+ | 0.264^**^ | (0.041) |  |  |
| Participation rate of younger workers in training |  |  | 0.597^**^ | (0.012) |

Notes: BIBB Training Panel 2024, N=1.310, Reported coefficients are average marginal effects; Standard errors in parentheses^, +^ *p* < 0.10, ^*^ *p* < 0.05, ^**^ *p* < 0.01, models include controls for company size, sector, West Germany, if the company provides apprenticeship training.

Table S9: Regression Results including Interaction Terms (different indicator on technology level (above overall average))

|  | Col1: Hiring  (#above average  technology usage  (overall average)) | | Col2: Training (#above average  technology usage  (overall average))) | |
| --- | --- | --- | --- | --- |
| Age-based stereotypes | -0.216^**^ | (0.079) | -0.237^**^ | (0.078) |
| Problems expected for most or all positions | 0.063 | (0.163) | 0.100 | (0.146) |
| Problems expected for some positions | -0.009 | (0.151) | 0.033 | (0.134) |
| No problems expected |  |  |  |  |
| Below average Tec usage (overall average) |  |  |  |  |
| Above average Tec usage (overall average) | 0.011 | (0.086) | -0.086 | (0.082) |
| Below average Tec usage (overall average)  # Age-based stereotypes |  |  |  |  |
| Above average Tec usage (overall average)  # Age-based stereotypes | 0.131 | (0.100) | 0.188^+^ | (0.107) |
| Qualification structure of older employees | -0.090 | (0.096) | 0.011 | (0.107) |
| Share of older employees within the organization | 1.381^**^ | (0.272) | -0.253 | (0.298) |
| Up to 20% of applicants aged 50+ |  |  |  |  |
| 21% to 50% of applicants aged 50+ | 0.604^**^ | (0.088) |  |  |
| More than 50% of applicants aged 50+ | 1.415^**^ | (0.182) |  |  |
| Participation rate of younger workers in training |  |  | 3.924^**^ | (0.145) |
| Pseudo *R*^2^ | 0.0588 |  | 0.2491 |  |

Notes: BIBB Training Panel 2024, N=1.310, Standardized beta coefficients; Standard errors in parentheses, ^+^ *p* < 0.10, ^*^ *p* < 0.05, ^**^ *p* < 0.01, models include controls for company size, sector, West Germany, if company provides apprenticeship training.

Table S9.1: Conditional effects of Age-based stereotypes: TEC (overall average)

|  | Col1: Hiring  (#above average  technology usage  (overall average)) | | Col2: Training  (#above average  technology usage  (overall average)) | |
| --- | --- | --- | --- | --- |
| Below average technology usage  (overall average) | -0.032^**^ | (0.012) | -0.036^**^ | (0.012) |
| Above average technology usage  (overall average) | -0.013 | (0.009) | -0.007 | (0.011) |

Notes: BIBB Training Panel 2024, N=1.310, Standard errors in parentheses, ^+^ *p* < 0.10, ^*^ *p* < 0.05, ^**^ *p* < 0

Table S10: Average Marginal Effects on hiring older employees (different indicators on type of technology)

|  | Col1:  AI usage  (yes/no) | | Col2:  ICT usage  (above average) | | Col3:  Non-ICT usage  (above average) | |
| --- | --- | --- | --- | --- | --- | --- |
| Age-based stereotypes | -0.022^**^ | (0.007) | -0.023^**^ | (0.007) | -0.022^**^ | (0.007) |
| Problems expected for most or all positions | 0.011 | (0.024) | 0.011 | (0.024) | 0.011 | (0.024) |
| Problems expected for some positions | -0.001 | (0.022) | -0.001 | (0.022) | -0.001 | (0.022) |
| No problems expected | 0.000 |  | 0.000 |  | 0.000 |  |
| No AI usage | 0.000 |  |  |  |  |  |
| AI usage | 0.002 | (0.015) |  |  |  |  |
| Below average ICT usage |  |  | 0.000 |  |  |  |
| Above average ICT usage |  |  | -0.002 | (0.013) |  |  |
| Below average Non-ICT usage (excl. AI) |  |  |  |  | 0.000 |  |
| Above average Non-ICT usage (excl. AI) |  |  |  |  | -0.001 | (0.013) |
| Qualification structure of older employees | -0.013 | (0.014) | -0.013 | (0.014) | -0.013 | (0.014) |
| Share of older employees within the organization | 0.205^**^ | (0.040) | 0.204^**^ | (0.040) | 0.204^**^ | (0.040) |
| Up to 20% of applicants aged 50+ | 0.000 |  | 0.000 |  | 0.000 |  |
| 21% to 50% of applicants aged 50+ | 0.093^**^ | (0.014) | 0.093^**^ | (0.014) | 0.093^**^ | (0.014) |
| More than 50% of applicants aged 50+ | 0.264^**^ | (0.041) | 0.264^**^ | (0.041) | 0.264^**^ | (0.041) |

Notes: BIBB Training Panel 2024, N=1.310, Reported coefficients are average marginal effects; Standard errors in parentheses^, +^ *p* < 0.10, ^*^ *p* < 0.05, ^**^ *p* < 0.01, models include controls for company size, sector, West Germany, if the company provides apprenticeship training.

Table S11: Average Marginal Effects on training older employees (different indicators on type of technology)

|  | Col1:  AI usage  (yes/no) | | Col2:  ICT usage  (above average) | | Col3:  Non-ICT usage  (above average) | |
| --- | --- | --- | --- | --- | --- | --- |
| Age-based stereotypes | -0.021^**^ | (0.008) | -0.021^**^ | (0.008) | -0.021^**^ | (0.008) |
| Problems expected for most or all positions | 0.018 | (0.022) | 0.017 | (0.022) | 0.017 | (0.022) |
| Problems expected for some positions | 0.007 | (0.020) | 0.007 | (0.020) | 0.007 | (0.020) |
| No problems expected | 0.000 |  | 0.000 |  | 0.000 |  |
| No AI usage | 0.000 |  |  |  |  |  |
| AI usage | -0.000 | (0.017) |  |  |  |  |
| Below average ICT usage |  |  | 0.000 |  |  |  |
| Above average ICT usage |  |  | -0.019 | (0.013) |  |  |
| Below average Non-ICT usage (excl. AI) |  |  |  |  | 0.000 |  |
| Above average Non-ICT usage (excl. AI) |  |  |  |  | 0.010 | (0.013) |
| Share of older employees within the organization | -0.037 | (0.045) | -0.042 | (0.045) | -0.035 | (0.045) |
| Participation rate of younger workers in training | 0.594^**^ | (0.012) | 0.597^**^ | (0.013) | 0.594^**^ | (0.013) |

Notes: BIBB Training Panel 2024, N=1.310, Reported coefficients are average marginal effects; Standard errors in parentheses^, +^ *p* < 0.10, ^*^ *p* < 0.05, ^**^ *p* < 0.01, models include controls for company size, sector, West Germany, if the company provides apprenticeship training.

Table S12: Regression Results including Interaction Terms (Hiring, different indicators on type of technology)

|  | Col1:  AI usage  (yes/no) | | Col2:  ICT usage  (above average) | | Col3:  Non-ICT usage  (above average) | |
| --- | --- | --- | --- | --- | --- | --- |
| Age-based stereotypes | -0.151^**^ | (0.056) | -0.212^*^ | (0.086) | -0.207^**^ | (0.063) |
| Problems expected for most or all positions | 0.072 | (0.162) | 0.064 | (0.163) | 0.059 | (0.161) |
| Problems expected for some positions | -0.004 | (0.151) | -0.010 | (0.151) | -0.010 | (0.150) |
| No problems expected | 0.000 |  | 0.000 |  | 0.000 |  |
| No AI usage | 0.000 |  |  |  |  |  |
| AI usage | 0.016 | (0.098) |  |  |  |  |
| No AI usage # Age-based stereotypes | 0.000 |  |  |  |  |  |
| AI usage # Age-based stereotypes | 0.004 | (0.113) |  |  |  |  |
| Below average ICT usage |  |  | 0.000 |  |  |  |
| Above average ICT usage |  |  | -0.006 | (0.089) |  |  |
| Below average ICT usage  # Age-based stereotypes |  |  | 0.000 |  |  |  |
| Above average ICT usage  # Age-based stereotypes |  |  | 0.108 | (0.105) |  |  |
| Below average Non-ICT usage (excl. AI) |  |  |  |  | 0.000 |  |
| Above average Non-ICT usage (excl. AI) |  |  |  |  | 0.007 | (0.085) |
| Below average Non-ICT usage (excl. AI)  # Age-based stereotypes |  |  |  |  | 0.000 |  |
| Above average Non-ICT usage (excl. AI)  # Age-based stereotypes |  |  |  |  | 0.177^+^ | (0.103) |
| Qualification structure of older employees | -0.089 | (0.096) | -0.086 | (0.095) | -0.090 | (0.095) |
| Share of older employees within the organization | 1.375^**^ | (0.270) | 1.364^**^ | (0.269) | 1.382^**^ | (0.271) |
| Up to 20% of applicants aged 50+ | 0.000 |  | 0.000 |  | 0.000 |  |
| 21% to 50% of applicants aged 50+ | 0.604^**^ | (0.088) | 0.605^**^ | (0.089) | 0.601^**^ | (0.088) |
| More than 50% of applicants aged 50+ | 1.409^**^ | (0.182) | 1.416^**^ | (0.182) | 1.424^**^ | (0.184) |
| Pseudo *R*^2^ | 0.0583 |  | 0.0586 |  | 0.0590 |  |

Notes: BIBB Training Panel 2024, N=1.310, Standardized beta coefficients; Standard errors in parentheses, ^+^ *p* < 0.10, ^*^ *p* < 0.05, ^**^ *p* < 0.01; models include controls for company size, sector, West Germany, if the company provides apprenticeship training.

Table S13: Regression Results including Interaction Terms (Training, different indicators on type of technology)

|  | Col1:  AI usage  (yes/no) | | Col2:  ICT usage  (above average) | | Col3:  Non-ICT usage  (above average) | |
| --- | --- | --- | --- | --- | --- | --- |
| Age-based stereotypes | -0.170^**^ | (0.056) | -0.256^**^ | (0.087) | -0.199^**^ | (0.064) |
| Problems expected for most or all positions | 0.107 | (0.145) | 0.098 | (0.147) | 0.104 | (0.146) |
| Problems expected for some positions | 0.040 | (0.133) | 0.032 | (0.134) | 0.039 | (0.134) |
| No problems expected | 0.000 |  | 0.000 |  | 0.000 |  |
| No AI usage | 0.000 |  |  |  |  |  |
| AI usage | -0.005 | (0.114) |  |  |  |  |
| No AI usage # Age-based stereotypes | 0.000 |  |  |  |  |  |
| AI usage # Age-based stereotypes | 0.189 | (0.146) |  |  |  |  |
| Below average ICT usage |  |  | 0.000 |  |  |  |
| Above average ICT usage |  |  | -0.120 | (0.087) |  |  |
| Below average ICT usage  # Age-based stereotypes |  |  | 0.000 |  |  |  |
| Above average ICT usage  # Age-based stereotypes |  |  | 0.196^+^ | (0.111) |  |  |
| Below average Non-ICT usage (excl. AI) |  |  |  |  | 0.000 |  |
| Above average Non-ICT usage (excl. AI) |  |  |  |  | 0.075 | (0.084) |
| Below average Non-ICT usage (excl. AI)  # Age-based stereotypes |  |  |  |  | 0.000 |  |
| Above average Non-ICT usage (excl. AI)  # Age-based stereotypes |  |  |  |  | 0.197^+^ | (0.111) |
| Qualification structure of older employees | 0.005 | (0.108) | 0.016 | (0.107) | -0.002 | (0.106) |
| Share of older employees within the organization | -0.230 | (0.299) | -0.270 | (0.298) | -0.213 | (0.300) |
| Participation rate of younger workers in training | 3.918^**^ | (0.146) | 3.920^**^ | (0.146) | 3.910^**^ | (0.146) |
| Pseudo *R*^2^ | 0.2485 |  | 0.2493 |  | 0.2489 |  |

Notes: BIBB Training Panel 2024, N=1.310, Standardized beta coefficients; Standard errors in parentheses, ^+^ *p* < 0.10, ^*^ *p* < 0.05, ^**^ *p* < 0.01; models include controls for company size, sector, West Germany, if the company provides apprenticeship training.

Table S12.1: Conditional effects of Age-based stereotypes: AI, ICT, Non-ICT (hiring)

|  | Col1: Hiring (#AI) |  | Col1: Hiring (#ICT) |  | Col1: Hiring (#Non-ICT) |  |
| --- | --- | --- | --- | --- | --- | --- |
| No AI usage | -0.023^**^ | (0.008) |  |  |  |  |
| AI usage | -0.022 | (0.015) |  |  |  |  |
| Below average ICT usage |  |  | -0.032^*^ | (0.013) |  |  |
| Above average ICT usage |  |  | -0.015^+^ | (0.009) |  |  |
| Below average Non-ICT usage (excl. AI) |  |  |  |  | -0.031^**^ | (0.009) |
| Above average Non-ICT usage (excl. AI) |  |  |  |  | -0.004 | (0.012) |

Notes: BIBB Training Panel 2024, N=1.310, Standard errors in parentheses, ^+^ *p* < 0.10, ^*^ *p* < 0.05, ^**^ *p* < 0.01

Table S13.1: Conditional effects of Age-based stereotypes: AI, ICT, Non-ICT (training)

|  | Col1:  Training (#AI) |  | Col2:  Training (#ICT) |  | Col2:  Training (#Non-ICT) |  |
| --- | --- | --- | --- | --- | --- | --- |
| No AI usage | -0.026^**^ | (0.008) |  |  |  |  |
| AI usage | 0.003 | (0.020) |  |  |  |  |
| Below average ICT usage |  |  | -0.039^**^ | (0.013) |  |  |
| Above average ICT usage |  |  | -0.009 | (0.010) |  |  |
| Below average Non-ICT usage (excl. AI) |  |  |  |  | -0.030^**^ | (0.010) |
| Above average Non-ICT usage (excl. AI) |  |  |  |  | -0.000 | (0.014) |

Notes: BIBB Training Panel 2024, N=1.310, Standard errors in parentheses, ^+^ *p* < 0.10, ^*^ *p* < 0.05, ^**^ *p* < 0.01

Table S14: Average Marginal Effects on hiring and training older employees (by firm size)

|  | Col1: Hiring  (small firms) | | Col2: Hiring  (large firms) | | Col3: Training (small firms) | | Col4: Training (large firms) | |
| --- | --- | --- | --- | --- | --- | --- | --- | --- |
| Age-based stereotypes | -0.037^**^ | (0.012) | -0.006 | (0.007) | -0.031^**^ | (0.012) | -0.008 | (0.010) |
| Problems expected for most or all positions | -0.003 | (0.036) | 0.030 | (0.024) | 0.034 | (0.030) | -0.013 | (0.032) |
| Problems expected for some positions | 0.008 | (0.034) | -0.007 | (0.022) | 0.010 | (0.028) | -0.010 | (0.028) |
| No problems expected |  |  |  |  |  |  |  |  |
| Below average technology usage |  |  |  |  |  |  |  |  |
| Above average technology usage | -0.021 | (0.022) | 0.011 | (0.012) | -0.014 | (0.018) | 0.009 | (0.016) |

Notes: BIBB Training Panel 2024, N=1.310 (small firms with up to 99 employees, N=658; large firms with 100 and more employees, N=652), Reported coefficients are average marginal effects; Standard errors in parentheses^, +^ *p* < 0.10, ^*^ *p* < 0.05, ^**^ *p* < 0.01; models include controls for sector, company size, West Germany, qualification level of older employees, the share of older employees in the company, and if the company provides apprenticeship training. Col1, Col2: models additionally control for the share of applications from older workers, Col3, Col4: models additionally control for the participation rate of employees below 50 years of age in further training.

Table S15: Average Marginal Effects on hiring and training older employees (by sector)

|  | Col1: Hiring  (services) | | Col2: Hiring  (manufacturing) | | Col3: Training  (services) | | Col4: Training (manufacturing) | |
| --- | --- | --- | --- | --- | --- | --- | --- | --- |
| Age-based stereotypes | -0.019^*^ | (0.009) | -0.029^*^ | (0.013) | -0.025^*^ | (0.010) | -0.018 | (0.013) |
| Problems expected for most or all positions | 0.001 | (0.029) | 0.024 | (0.045) | 0.017 | (0.026) | 0.011 | (0.042) |
| Problems expected for some positions | -0.005 | (0.027) | 0.005 | (0.040) | 0.012 | (0.025) | -0.016 | (0.036) |
| No problems expected |  |  |  |  |  |  |  |  |
| Below average technology usage |  |  |  |  |  |  |  |  |
| Above average technology usage | 0.001 | (0.016) | -0.022 | (0.022) | -0.035^*^ | (0.016) | 0.052^**^ | (0.020) |

Notes: BIBB Training Panel 2024, N=1.310 (firms belonging to the services sector, N=859; firms belonging to the manufacturing sector, N=451), Reported coefficients are average marginal effects; Standard errors in parentheses^, +^ *p* < 0.10, ^*^ *p* < 0.05, ^**^ *p* < 0.01; models include controls for sector, company size, West Germany, qualification level of older employees, the share of older employees in the company, and if the company provides apprenticeship training. Col1, Col2: models additionally control for the share of applications from older workers, Col3, Col4: models additionally control for the participation rate of employees below 50 years of age in further training.

Table S16: Regression Results including Interaction Terms (firm size, sector)

|  | Col1: Hiring  (#firm size) | | Col2: Training  (#firm size) | | Col3: Hiring  (#sector) | | Col4: Training  (#sector) | |
| --- | --- | --- | --- | --- | --- | --- | --- | --- |
| Age-based stereotypes | -0.043 | (0.053) | -0.059 | (0.073) | -0.202^*^ | (0.085) | -0.103 | (0.097) |
| 100 and more employees |  |  |  |  |  |  |  |  |
| 1 to 99 employees | 0.293^**^ | (0.088) | 0.172^+^ | (0.088) |  |  |  |  |
| 100 and more employees  # Age-based stereotypes |  |  |  |  |  |  |  |  |
| 1 to 99 employees  # Age-based stereotypes | 0.195^*^ | (0.093) | 0.138 | (0.103) |  |  |  |  |
| Problems expected for most or all positions | 0.022 | (0.162) | 0.110 | (0.146) | 0.067 | (0.162) | 0.116 | (0.146) |
| Problems expected for some positions | -0.074 | (0.150) | 0.038 | (0.134) | -0.009 | (0.151) | 0.047 | (0.134) |
| No problems expected |  |  |  |  |  |  |  |  |
| Below average technology usage |  |  |  |  |  |  |  |  |
| Above average technology usage | -0.070 | (0.086) | -0.034 | (0.083) | -0.045 | (0.087) | -0.035 | (0.082) |
| Manufacturing and Industry |  |  |  |  |  |  |  |  |
| Service and Public Sector | -0.021 | (0.089) | 0.038 | (0.087) |  |  |  |  |
| Manufacturing and Industry |  |  |  |  |  |  |  |  |
| Service and Public Sector |  |  |  |  | -0.042 | (0.088) | 0.035 | (0.087) |
| Manufacturing and Industry  # Age-based stereotypes |  |  |  |  |  |  |  |  |
| Service and Public Sector  # Age-based stereotypes |  |  |  |  | 0.074 | (0.106) | -0.050 | (0.116) |
| 1 to 19 employees |  |  |  |  |  |  |  |  |
| 20 to 99 employees |  |  |  |  | -0.591^**^ | (0.167) | -0.057 | (0.166) |
| 100 to 199 employees |  |  |  |  | -0.773^**^ | (0.173) | -0.230 | (0.181) |
| 200 or more employees |  |  |  |  | -0.782^**^ | (0.171) | -0.213 | (0.182) |
| Pseudo *R*^2^ | 0.0540 |  | 0.2485 |  | 0.0585 |  | 0.2482 |  |

Notes: BIBB Training Panel 2024, N=1.310, Standardized beta coefficients; Standard errors in parentheses, ^+^ *p* < 0.10, ^*^ *p* < 0.05, ^**^ *p* < 0.01; models include controls for West Germany, qualification level of older employees, the share of older employees in the company, and if the company provides apprenticeship training. Col1, Col3: models additionally control for the share of applications from older workers, Col2, Col4: models additionally control for the participation rate of employees below 50 years of age in further training.

Table S16.1: Conditional effects of Age-based stereotypes by FIRM SIZE and SECTOR

|  | Col1: Hiring  (#Firm size) | | Col2: Training  (#Firm size) | | Col3: Hiring  (#Sector) | | Col4: Training  (#Sector) | |
| --- | --- | --- | --- | --- | --- | --- | --- | --- |
| 100 and more employees | -0.006 | (0.007) | -0.009 | (0.011) |  |  |  |  |
| 1 to 99 employees | -0.038^**^ | (0.013) | -0.030^**^ | (0.011) |  |  |  |  |
| Manufacturing and Industry |  |  |  |  | -0.031^*^ | (0.013) | -0.016 | (0.015) |
| Services and Public Sector |  |  |  |  | -0.019^*^ | (0.009) | -0.023^*^ | (0.009) |

Notes: BIBB Training Panel 2024, N=1.310, Standard errors in parentheses, ^+^ *p* < 0.10, ^*^ *p* < 0.05, ^**^ *p* < 0.01

Table S17: Regression Results including Interaction Terms (above average share of older employees within firm)

|  | Col1: Hiring  (# above average share  of older employees  within firm) | |
| --- | --- | --- |
| Age-based stereotypes | -0.153^*^ | (0.072) |
| Below average share of older employees within firm |  |  |
| Above average share of older employees within firm | 0.389^**^ | (0.087) |
| Below average share of older employees within firm  # Age-based stereotypes |  |  |
| Above average share of older employees within firm  # Age-based stereotypes | -0.013 | (0.100) |
| Problems expected for most or all positions | 0.061 | (0.161) |
| Problems expected for some positions | -0.017 | (0.149) |
| No problems expected |  |  |
| Below average technology usage |  |  |
| Above average technology usage | -0.046 | (0.087) |
| Qualification structure of older employees | -0.096 | (0.095) |
| Up to 20% of applicants aged 50+ |  |  |
| 21% to 50% of applicants aged 50+ | 0.632^**^ | (0.088) |
| More than 50% of applicants aged 50+ | 1.443^**^ | (0.181) |
| Pseudo *R*^2^ | 0.0571 |  |

Notes: BIBB Training Panel 2024, N=1.310, Standardized beta coefficients; Standard errors in parentheses, ^+^ *p* < 0.10, ^*^ *p* < 0.05, ^**^ *p* < 0.01, models include controls for company size, sector, West Germany, if the company provides apprenticeship training.

Table S17.1: Conditional effects of Age-based stereotypes: ABOVE AVERAGE SHARE OF OLDER EMPLOYEES WITHIN FIRM (hiring)

|  | Col1: Hiring  (#above average share  of older employees  within firm) | |
| --- | --- | --- |
| Below average share of older employees within firm | -0.021^*^ | (0.010) |
| Above average share of older employees within firm | -0.028^*^ | (0.012) |

Notes: BIBB Training Panel 2024, N=1.310, Standard errors in parentheses, ^+^ *p* < 0.10, ^*^ *p* < 0.05, ^**^ *p* < 0.01
